# Patterns of alteration in boar semen quality from 9 to 37 months old and improvement by protocatechuic acid

**DOI:** 10.1186/s40104-024-01031-6

**Published:** 2024-05-17

**Authors:** Ruizhi Hu, Xizi Yang, Jiatai Gong, Jing Lv, Xupeng Yuan, Mingkun Shi, Chenxing Fu, Bie Tan, Zhiyong Fan, Liang Chen, Hongfu Zhang, Jianhua He, Shusong Wu

**Affiliations:** 1 Hunan Collaborative Innovation Center for Utilization of Botanical Functional Ingredients, College of Animal Science and Technology, Hunan Agricultural University, Changsha, 410128 China; 2 College of Animal Science and Technology, Hunan Biological and Electromechanical Polytechnic, Changsha, 410127 China; 3 State Key Laboratory of Animal Nutrition, Institute of Animal Sciences, Chinese Academy of Agricultural Sciences, Beijing, 100081 China

**Keywords:** Antioxidants, Boars, Protocatechuic acid, Semen quality, Signaling pathway

## Abstract

**Background:**

Comprehending the patterns of alteration in boar semen quality and identifying effective nutritional interventions are crucial for enhancing the productivity of commercial pig systems. This study aimed to examine the alteration in semen quality in boars, and assess the impact of protocatechuic acid (PCA) on semen quality during the phase of declining semen quality.

**Methods:**

In Exp. 1, a total of 38 Pig Improvement Company (PIC) boars were selected and their semen quality data were recorded from the age of 9 to 37 months. In Exp. 2, 18 PIC boars (28 months old) were randomly assigned into three groups (*n* = 6) and fed a basal diet, a basal diet containing 500 or 1,000 mg/kg PCA, respectively. The experiment lasted for 12 weeks.

**Results:**

The semen volume, concentration, and total number of spermatozoa in boars exhibited an increase from 9 to 19 months old and showed a significant linear decreased trend in 28, 24, and 22 months old. Sperm motility displayed an upward trajectory, reaching its peak at 20 months of age, and showed a significant linear decreased trend at 20 months old. Dietary supplementation of PCA demonstrated an effect to mitigate the decrease in semen volume, concentration of spermatozoa, total number of spermatozoa (*P* > 0.05), and significantly increased the sperm motility (*P* < 0.05). Moreover, supplementation of 1,000 mg/kg PCA significantly increased the sperm viability (*P* < 0.05). Analysis on cellular signaling pathways revealed that PCA restored serum testosterone levels and alleviated oxidative damage by upregulating the expression of HO-1, SOD2, and NQO1 in testicular stromal cells. Notably, PCA can enhance phosphorylation by selectively binding to AMP-activated protein kinase (AMPK) protein, thereby improving sperm mitochondrial function and augmenting sperm motility via *PGC-1*/*Nrf1*.

**Conclusions:**

These data elucidated the pattern of semen quality variation in boars within the age range of 9 to 37 months old, and PCA has the potential to be a natural antioxidant to enhance sperm quality through modulation of the AMPK/PGC-1/Nrf1 signaling pathway.

**Supplementary Information:**

The online version contains supplementary material available at 10.1186/s40104-024-01031-6.

## Introduction

The boars are a fundamental and crucial aspect of the pig industry. The advancement of the large-scale pig industry has led to the rapid promotion and application of pig artificial insemination technology. As the number of breeding boars in production diminishes, its significance is increasingly pronounced. The shortened reproductive lifespan of boar is a significant concern. In the 1990s, the reproductive lifespan of boars employed for breeding purposes was constrained to a mere 20 months [1]. In the early twentieth century, the age at which boars were culled predominantly revolved around 24 months [2]. However, recent investigations have revealed that the disposal of breeding boars is predominantly transpiring at 18 months [3]. This observation implies that as advancements continue, the utilization duration for breeding boars is progressively diminishing. Furthermore, due to the widespread utilization of artificial insemination techniques, a considerable amount of research has primarily focused on semen storage. However, there is limited scientific investigation regarding extending the utilization period for breeding boars and the enhancement of semen quality.

Under natural conditions, the peak reproductive capacity of boars is typically observed around 18 months of age. Although theoretically the utilization period of breeding boars can be extended up to 20 months, in practical production settings, boars are typically retired at a much younger age [3, 4]. Insufficient nutrition during the breeding period has been shown to results in an increase in the production of abnormal sperm and a decline in mating capacity [5]. Therefore, contemporary nutritional regimens strive to provide higher levels of nourishment for boars during the mating season, which result in excessive weight gain and a shortened lifespan for breeding boars.

Several factors, including elevated circulating endotoxins, reduced testosterone levels, and oxidative stress, can negatively impact the semen quality of boars, leading to decreased sperm production and vitality [6–8]. Testosterone, which is present in high levels within the testes, plays a crucial role in spermatogenesis. Research indicates elevated serum endotoxin levels are inversely correlated with testosterone levels [9]. Even low-dose endotoxin exposure can trigger acute inflammatory responses, leading to impaired testicular function in males and accompanied by reduced testosterone levels [10]. The motility and vitality of sperm are critical determinants of successful mating and fertilization. However, sperm cells are highly susceptible to the damaging effects of reactive oxygen species (ROS) [11]. The polyunsaturated fatty acids that constitute the sperm cell membrane are particularly vulnerable to ROS attack, resulting in cellular damage [12]. Sperm vitality relies on the functionality of the flagellum, mitochondria are densely distributed in the middle region of the sperm flagellum, which provide the necessary energy for sperm motility [13]. Although mitochondria are the primary source of ROS generation within cells, they are also prone to ROS attack, leading to mitochondrial dysfunction and subsequent reduction in sperm vitality [14].

Protocatechuic acid (PCA) is a bioactive phenolic metabolite of polyphenol cyanidin-3-O-glucoside (C3G). We have reported PCA is the main metabolite of C3G, which has lipid-lowering, antioxidant, and anti-inflammatory effects [15, 16]. In the follow-up study, we found that PCA also has protective effects on intestinal barrier function by regulating intestinal flora, enhancing the relative abundance of SCFA-producing floras [17]. The objective of this study is to analyze the evolution of semen quality in boars from the introduction stage to 37 months of age, identify the specific stage at which semen quality begins to decline, and investigate the potential benefits of PCA in enhancing semen quality during this period.

## Materials and methods

### Experimental design

In Exp. 1, a total of 38 Pig Improvement Company (PIC) boars were selected and their semen quality data were recorded from the age of 9 months. The boars were managed according to the standard guidelines outlined in the PIC Boars Station management manual. In Exp. 2, 18 28-month-old PIC boars with similar body weight and semen quality (following 4 consecutive semen collections) were randomly allocated into 3 groups using Block Randomization scheme (www.randomiser.org), each consisting of 6 boars. The boars were fed a basal diet, a basal diet supplemented with 500 mg/kg PCA, and a basal diet supplemented with 1,000 mg/kg PCA, respectively. The experiment lasted for a duration of 12 weeks. The risk of bias (RoB) was described and judged according to the SYRCLEs RoB tool [18].

The feeding mode utilized is single pen feeding, with the pig house being cooled by a water curtain and heated by floor heating. The temperature within the pig house is maintained at 21 °C, while humidity are controlled at 50%. Ventilation within the pig house is ensured, with an air flow rate of 600 ft/min. Additionally, the ammonia content of the air is kept below 14 mg/m^3^, carbon dioxide content below 5,890 mg/m^3^, and hydrogen sulfide content below 7 mg/m^3^. Bedding is changed twice a week, and mycotoxin detection is conducted on the pig house environment on a monthly basis. The frequency of sperm collection in boars aged over 12 months is typically 3–4 times every 2 weeks. All pigs have ad libitum access to water and are fed twice daily at 7:00 and 14:00. The basal diets of boars were formulated according to NRC (2012) [19]. The ingredients and nutrient composition of the basal diets were shown in Table 1. Weekly recordings were made of semen collection, sperm motility, sperm density, and total sperm count. At the end of the 12-week period, semen samples were collected for further analysis. Blood samples were collected and serum was obtained through centrifugation at a speed of 1,500 × *g* for 10 min.

**Table 1:** Ingredients and nutrient composition of the basal diet (as fed basis)

Ingredients	%
Corn	47.00
Flour	20.00
Soybean meal (43% CP)	9.00
Fermented soybean meal	5.00
Extruded soybean	3.00
Fish meal	3.00
Wheat bran	10.00
Soybean oil	1.00
Premix^a^	2.00
Nutrient composition (calculated value)	
DE, kcal/kg	3,360
NE, kcal/kg	2,400
CP	17.00
EE	4.50
CF	3.65
NDF	11.80
SID Lys	0.96
SID Met	0.32
SID Thr	0.54
SID Ser	0.67
Total P	0.65
Digestible P	0.34
Total calcium	0.65

^a^Premix provided per kilogram of complete feed: Cu 15 mg/kg, Fe 250 mg/kg, Zn 60 mg/kg, Mn 70 mg/kg, I 0.6 mg/kg, VA 10,000 IU, VD 3,000 IU, riboflavin 4.0 mg, pantothenic acid 40 mg, nicotinic acid 40 mg, biotin 0.3 mg, folic acid 1.0 mg, VB_12_ 0.04 mg

### Semen analysis

Sperm volume was measured using graduated tubes. Concentration of spermatozoa was measured by the sperm quality detection system (SDM1, Minitube, Germany), and each sample was repeated for 3 times, and the average value was calculated. Total number of spermatozoa = Sperm volume × Concentration of spermatozoa. Sperm motility and viability were then analyzed by computer assisted sperm analysis. Briefly, 10 μL of the incubated semen was put in a disposable analysis chamber slide (ML-CASA10-4, Mailang, Nanning, China) and maintained at 37 °C, and 5 fields were selected for the analysis. The computer assisted sperm analysis captures each filed of sperm for 2 s videos, then analyzed at 25 frames/s. The analyzed variables included the percentage of spermatozoa that showed movement with straightness (STR) > 75% within sample, curvilinear velocity (VCL, μm/s), straight line velocity (VSL, μm/s), average path velocity (VAP, μm/s), straightness (STR = VSL/VAP × 100), linearity of forward progression (LIN = VSL/VCL × 100), wobble (WOB = VAP/VCL × 100), amplitude of lateral head displacement (ALH, µm) and beat-cross frequency (BCF, Hz). The sperm viability = (VSL × 0.895) + (VAP × 0.686) + (VCL × 0.895) + (ALH × 0.592) × 100%. Sperm with an average path velocity (VAP; µm/s) higher than 10 μm/s were considered motile, and those with an index of straightness (STR; straight-line velocity (VSL)/VAP; %) higher than 45% were classified as progressively motile. The sperm motility = (Motile sperm count/Total sperm count) × 100%.

### ROS analysis

The sperm sample underwent centrifugation at 500 × *g* for 5 min, followed by three washes with pre-cooled sterile PBS solution. Subsequently, the semen was diluted to a concentration of 1 × 10^5^/mL, applied to a slide, and allowed to dry. The OxiSelect Intracellular ROS Assay Kit from Cell Biolabs was utilized to introduce ROS probes, with excitation at 488 nm and emission at 525 nm. Fluorescence microscopy was employed to capture images. The sperm sample underwent centrifugation at 500 × *g* for 5 min, followed by three washes with pre-cooled sterile PBS solution. Subsequently, the semen was diluted to a concentration of 1 × 10^7^/mL into 1.5-mL centrifuge tube, then the OxiSelect Intracellular ROS Assay Kit from Cell Biolabs was utilized to introduce ROS probes and the fluorescence intensity of ROS in tube was measured by a porous chemiluminescence detector (Varioskan Flash, USA).

### Testosterone levels

Testosterone levels in the TM3 cell supernatant were measured using the Testosterone Parameter Assay Kit (KGE010 R&D Systems Inc., Minneapolis, MN, USA). Serum testosterone levels were measured using the Testosterone Parameter Assay Kit (MM 0410O2 MEIMIAN, Inc., Yancheng, China).

### Real-time PCR

The mRNA expression was determined by real-time PCR as pervious study [20, 21]. The mRNA was extracted from sperm with Total RNA kit (Steadypure universal RNA ectraction kit, Accurate Biotechnology Co., Ltd. Changsha, Hunan, China) according to the manufacturer’s instructions. The concentration of RNA was quantified using a NanoDrop^®^ lite (Thermo Fisher, USA). Reverse transcription of 1 μg total RNA was performed by reverse transcription kit (Evo M-MLV RT Premix, Accurate Biotechnology Co., Ltd.). The PCR reactions were performed in a 20 μL total reaction volume, which included 10 μL of 2 × SybrGreen qPCR Master Mix (SYBR^®^ Green Premix Pro Taq HS qPCR kit), 0.4 μL each of the forward and reverse primers (10 μmol/L), 2 μL of cDNA template, and 7.2 μL of sterilized water. The PCR was carried out on a LightCycler480 Real-Time PCR system (Rotkreuz, Switzerland). The thermal cycler parameters were as follows: 3 min at 95 °C, 40 cycles for 5 s at 95 °C, 30 s at 60 °C. The stability of the β-actin and *GADPH* genes was evaluated by measuring the fluctuation range of the Ct values. The 2^−ΔΔCT^ method was used for data analysis. Primers used in this study are shown in Table S1.

### Cell culture

TM3 cells were cultured at 37 °C in a 5% CO_2_ atmosphere in DMEM/F12 containing 10% fetal bovine serum. TM3 cells (1 × 10^6^) were seeded in 6 cm dishes, after incubating for 24 h, the cells were starved by being cultured in serum-free for another 2.5 h to eliminate the influence of FBS. The cells were pretreated with 30, 60, 90 μmol/L PCA for 30 min. HO-1, NQO1 and SOD2 were detected after 12 h.

### Cell viability

The survival rate of TM3 cells was assessed using the CCK8 method. A cell suspension containing 1,000 cells per well was incubated overnight on a 96-well plate. After a 30-min treatment with various concentrations of PCA, 15 μmol/L 2,2′-Azobis (2-methylpropionamidine) dihydrochloride (AAPH) was introduced to each well. Following a 12-h incubation period, 10 μL of CCK8 solution was added to each well and the plate was placed in a 37 °C and 5% CO_2_ incubator for 2 h. The absorbance value was measured at a wavelength of 545 nm to calculate the survival rate.

### Transmission electron microscopy mitochondrial ultrastructure

The semen sample was rinsed three times with 0.1 mol/L phosphate buffer, fixed with 2% osmic acid, and rinsed again with phosphate buffer. Following the rinsing process, the samples underwent dehydration, permeation, and embedding. The slices were accurately positioned using an ultra-thin microtome. After being stained with 2% uranium acetate and lead citrate, the slices were air-dried overnight at room temperature. The sperm mitochondria were then examined and imaged using transmission electron microscopy (Hitachi H‐7700; Hitachi High Technologies Corp.).

### ATP level

The ATP level of sperm was quantified using the established protocol of the ATP content assay kit (Cat. #AKOP004M, Boxbio, China). Sperm were adjusted to a concentration of 1 × 10^9^ sperm/mL following PBS washing centrifugation and subsequently homogenized in ATP extraction buffer. The resulting homogenate was subjected to centrifugation at 10,000 × *g* for 10 min, and the resulting supernatant was collected and subjected to chloroform extraction. A 200 μL aliquot of the extract was utilized for ATP content determination. The sample, along with a standard sample (0.625 μmol/mL ATP in ddH_2_O), was incubated with a working buffer containing hexokinase, glucose, glucose dehydrogenase, and NADP. The absorbance of the synthesized NADPH was measured at 340 nm by an enzyme-labeler at two time points: 10 s and 190 s after incubation. The ATP content of the sample (expressed in μmol/10^9^) was determined by comparing it with a standard sample.

### Mitochondrial membrane potential

The mitochondrial membrane potential of sperm was assessed using the JC-1 fluorescence method. Following washing of the sperm with PBS, 10 μL sperm suspension was obtained and mixed with 6.8 μL of JC-1 staining solution. The mixture was then incubated in a wet box at a constant temperature of 37 °C for 30 min, while being shielded from light. Subsequently, 10 μL solution of PI was added and incubated for an additional 5 min in the absence of light. The resulting supernatant was centrifuged and resuspended in PBS. The sperm density was adjusted to 5 × 10^6^ sperm/mL, and the JC-1 monomer was detected using an excitation wavelength of 490 nm and an emission wavelength of 530 nm, and the JC-1 polymer was identified through the utilization of a 525 nm excitation wavelength and a 590 nm emission wavelength. The fluorescence intensity of JC-1 polymer and JC-1 monomer was measured by a porous chemiluminescence detector (Varioskan Flash, USA).

### Semen mitochondrial DNA quantification

To determine the quantity of mitochondria, the mitochondrial DNA was measured through quantitative polymerase chain reaction (qPCR). The extraction of semen genomic DNA was performed using Qiagen DNA extraction kits (Qiagen, Hilden, Germany). The mitochondrial encoded NADH dehydrogenase-1 (*ND-1*) was employed as a mitochondrial marker gene, while the glucagon gene (GCG) served as the internal reference gene.

### Western bolt

Western blotting, in brief [22], samples were accurately weighed (< 100 mg), RIPA lysis buffer were added according to mass:volume = 1:9 and homogenized with low temperature, centrifuged at 12,000 × *g* for 10 min at 4 °C, then the protein was denatured and the protein concentration was adjusted. Run on different concentrations of SDS-PAGE as needed, and then transfer to PVDF membrane. The blotted membranes were incubated with specific primary antibodies overnight at 4 °C and further incubated with HRP bound secondary antibodies for 1 h. Binding antibodies were detected and further quantified. The bound antibodies were then detected by GE ImageQuant LAS 4000. the references of antibodies and final concentrations during incubations used in this study are shown in Table S2.

### Pull-down assay

PCA-Sepharose 4B beads were prepared as previous studies [23]. Briefly, weighted 5 mg PCA into a 1.5-mL tube, pre-dissolved with a proper amount of DMSO. Then coupled to CNBr-activated Sepharose 4B beads in coupling buffer overnight at 4 °C. The mixture was centrifuged at 1,000 × *g* for 3 min at 4 °C and then removed the supernatant, added coupling buffer at a volume of beads × 5, centrifuged at 1,000 × *g* for 3 min at 4 °C, removed the supernatant, added 0.1 mol/L Tris–HCl (pH 8.0) at a volume of beads volume × 5, for 2 h rotation at room temperature. After washing three times with 0.1 mol/L acetate buffer (pH 4.0) containing 0.5 mol/L NaCl, the mixture was further washed with 0.1 mol/L Tris–HCl (pH 8.0) buffer containing 0.5 mol/L NaCl. The uterus samples of mice lysed in advance (500 g/mL) was incubated at 4 °C overnight with Sepharose 4B beads or Sepharose 4B PCA-coupled beads (100 μL, 50% slurry) in reaction buffer (NP-40). The beads were then washed five times with a washing buffer. The proteins bound to the beads were detected by Western blotting with each specific antibody.

### Molecular modeling

The modeling of PCA to PI3K proteins (3HIZ) was performed using Molecular Operating Environment software (MOE, Version 2019). Hydrogen atoms were first added, and force field atomic charges were assigned. Docking of PCA to protein was done using MOE-ASEDock 2019 software.

### Statistical analysis

The data were arranged using Excel (version 2019). Statistical analyses were performed using SPSS v19.0 (SPSS, Inc.). The graphs were performed using GraphPad Prism version 8.0 (GraphPad Software, San Diego, CA, USA). In Exp. 1, the correlation between month-old and semen quality was performed using simple linear regression model with least square method, the differences between two groups were analyzed by independent samples *t*-test, the one-way analysis of variance using the general linear model procedure for multiple group comparisons. In Exp. 2, one-way analysis of variance (one-way ANOVA) was used for multiple group comparisons. The data are presented as mean ± SD, *P* < 0.05 was considered significant, *P* < 0.01 was considered extremely significant.

## Results

### Patterns of semen quality changes in boars from 9 to 28 months of age

**Fig. 1:**
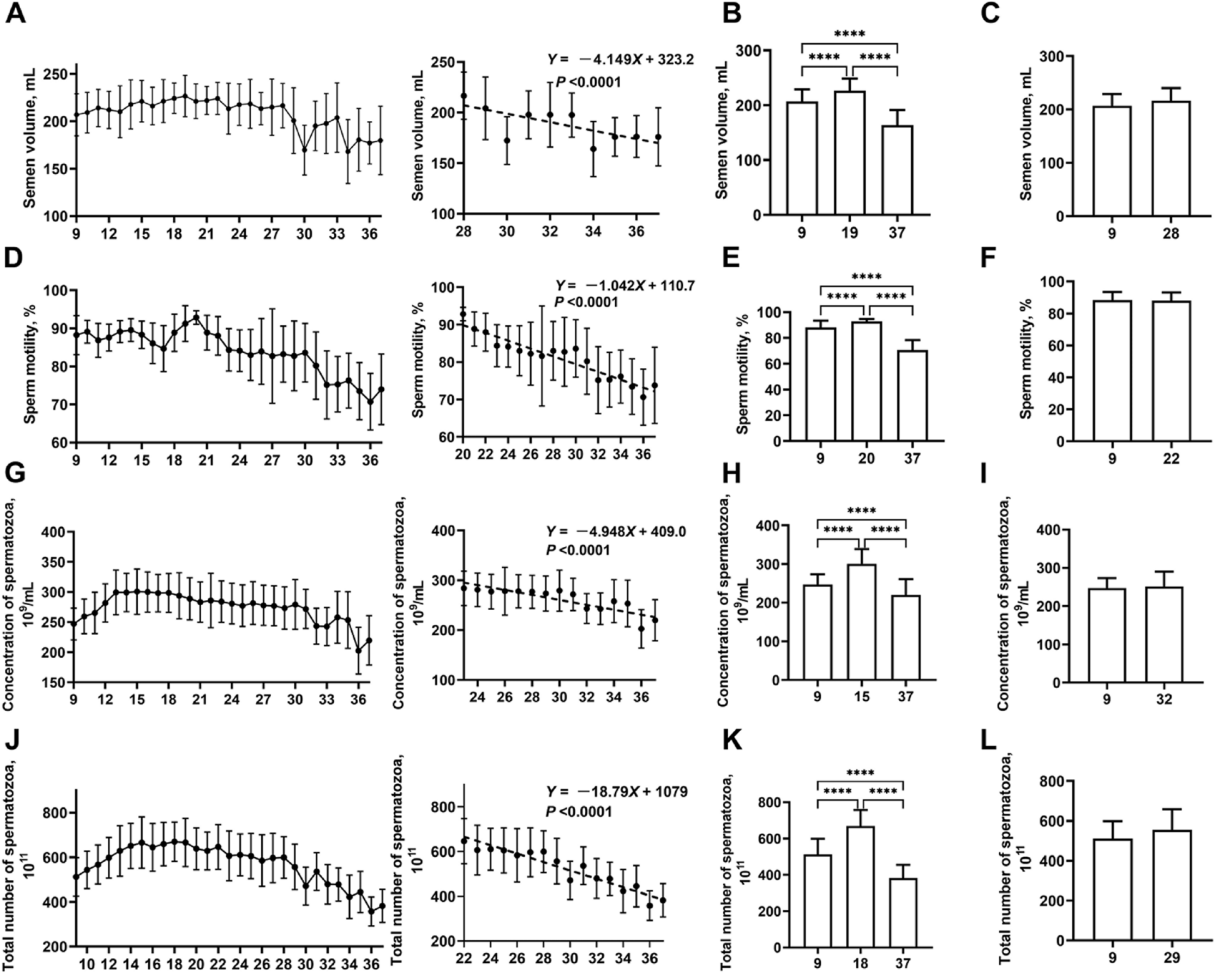
The temporal variations in the quality of boar semen across the age range of 9 to 37 months. The variations in semen volume (**A**), sperm motility (**D**), concentration of spermatozoa (**G**), and total number of spermatozoa (**J**) in boars aged 9–37 months. Semen volume in 9, 19, and 37 months old (**B**). Sperm motility in 9, 20, and 37 months old (**E**). Concentration of spermatozoa in 9, 15, and 37 months old (**H**). Total number of spermatozoa in 9, 18, and 37 months old (**K**). Semen volume in 9 and 28 months old (**C**). Sperm motility in 9 and 22 months old (**F**). Concentration of spermatozoa in 9 and 32 months old (**I**). Total number of spermatozoa in 9 and 29 months old (**L**). ^****^P < 0.0001

The patterns of semen quality changes in boars from 9 to 37 months old are illustrated in Fig. 1. The semen volume gradually increased from 9 to 19 months old, significantly rising from 206 mL to a peak of 226 mL (*P* < 0.0001) (Fig. 1A and B). From 28 months old, semen volume followed linear decrease. Subsequently, the semen volume at 28 months old was consistent with 9 months old (Fig. 1C), reaching a significant decrease to 164 mL at 37 months old (*P* < 0.001) (Fig. 1A and B). Sperm motility demonstrated fluctuating trends from 9 to 20 months old, with over 85% motility observed (Fig. 1D). The peak semen motility of 90.7% was reached at 20 months old. From 20 months old, semen motility followed linear decreasing trajectories (*P* < 0.001) (Fig. 1D and E). Subsequently, the semen motility in 22-month-old was consistent with 9-month-old, and significantly reduced to 70.7% at 37-month-old (*P* < 0.0001) (Fig. 1E and F). Concentration of spermatozoa gradually increased from 9 to 15 months old, significantly rising from 243 × 10^9^/mL to a peak of 298 × 10^9^/mL (*P* < 0.0001) (Fig. 1G). From 24-month-old, concentration of spermatozoa followed linear decreasing trajectories (*P* < 0.001) (Fig. 1G). Subsequently, the concentration of spermatozoa at 32-month-old was consistent with 9-month-old, and reached a significant decrease of 219 × 10^9^/mL at 37-month-old (*P* < 0.0001) (Fig. 1H and I). Total number of spermatozoa showed a progressive increase from 9 to 18 months old, significantly increased from 512 × 10^11^ to a peak of 669 × 10^11^ (*P* < 0.0001) (Fig. 1J). From 22 months old, the total number of spermatozoa followed linear decreasing trajectories (*P* < 0.001) (Fig. 1J). Subsequently, the total number of spermatozoa in 29-month-old was consistent with 9-month-old, reaching a significant decrease of 381 × 10^11^ at 37-month-old (*P* < 0.0001) (Fig. 1K and L).

### Protocatechuic acid alleviate the decline in semen quality and enhance sperm vitality

**Fig. 2:**
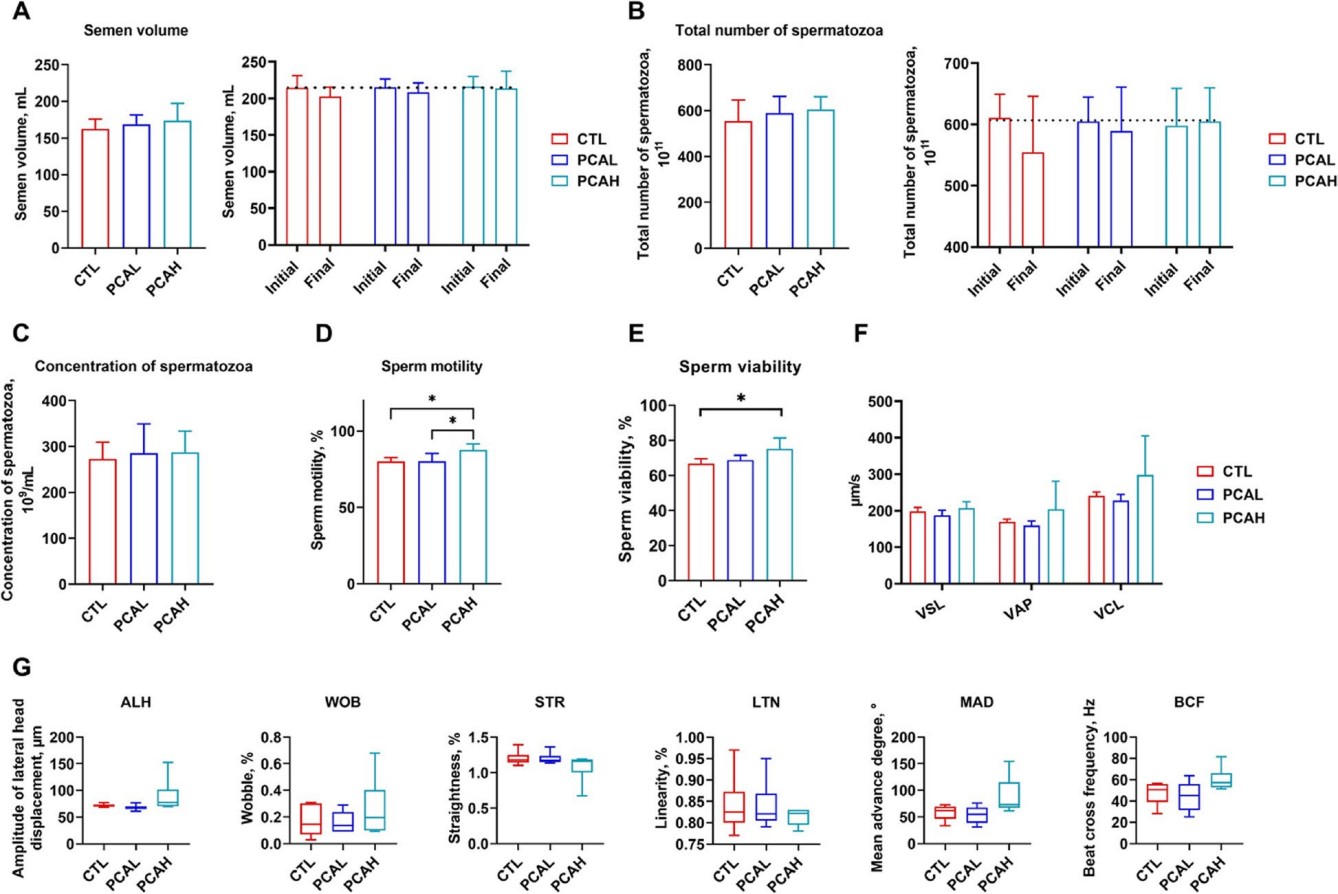
Effect of PCA on the semen quality of boars. Effect of protocatechuic acid (PCA) on the semen volume (**A**), total number of spermatozoa (**B**), concentration of spermatozoa (**C**), sperm motility (**D**) and sperm viability (**E**) in 28-mouth-old boars. Effect of PCA on the sperm motility parameters in 28-mouth-old boars (**F** and **G**). *VSL* Straight-line velocity, *VAP* Path velocity, *VCL* Curvilinear velocity, *ALH* Amplitude of lateral head displacement, *WOB* Wobble, *LTN* Linearity, *STR* Percentage straightness, *CTL* Control group, *PCAL* Basic diet containing 500 mg/kg PCA group, *PCAH* Basic diet containing 1,000 mg/kg PCA group. Data were shown as means ± SD (*n* = 6). ^*^P < 0.05, ^**^P < 0.01, ^***^P < 0.001, ^****^P < 0.0001

PCA, a dietary polyphenol, was added to the boars' daily diet at 500 and 1,000 mg/kg. After a 12-week feeding period, as shown in Fig. 2, dietary supplementation of PCA resulted in improved semen volume and total number of spermatozoa compared to the control group (*P* > 0.05) (Fig. 2A and B). It mitigated the decline in semen volume and total number of spermatozoa, with the PCAH group exhibiting similar values as those at the beginning of the experiment (Fig. 2A and B). Dietary supplementation of PCA showed no significant effect on concentration of spermatozoa (Fig. 2C). Furthermore, the addition of 1,000 mg/kg PCA significantly increased sperm motility (*P* < 0.05) (Fig. 2D). Compared to the control group, the addition of 1,000 mg/kg PCA significantly improved sperm vitality (*P* < 0.05) and enhanced parameters such as VSL, VAP, VCL, WOB, BCF, and MAD (*P* > 0.05) (Fig. 2E and G).

### Protocatechuic acid restores testosterone secretion by increasing the expression of antioxidant enzymes

**Fig. 3:**
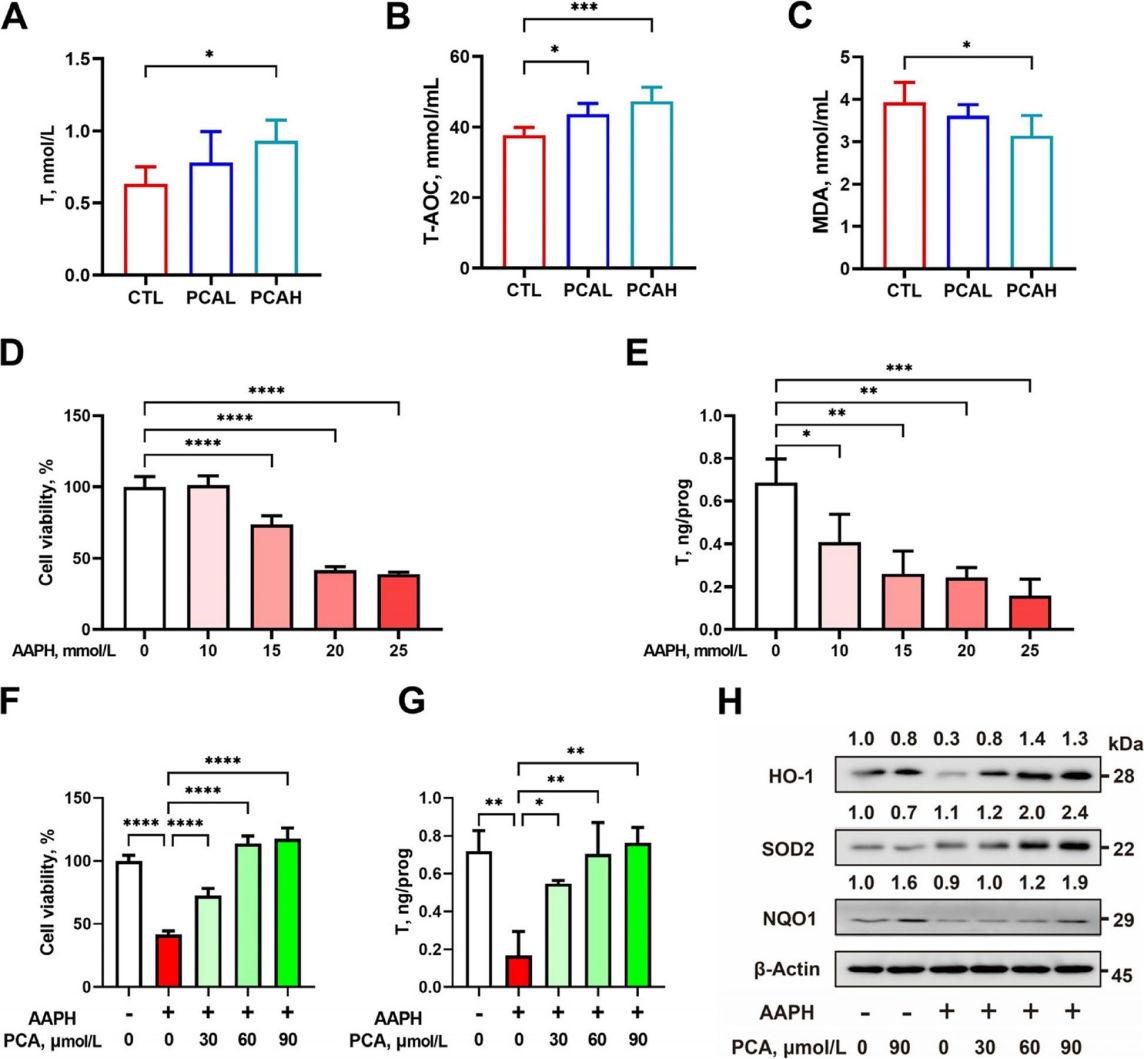
Protocatechuic acid (PCA) ameliorates the impairment of testicular interstitial cells through enhancement of the antioxidant capacity. The impact of PCA on serum testosterone levels (**A**), antioxidant capacity (**B**), and malondialdehyde (**C**) in boars. The impact of AAPH on survival rate (**D**) and expression of restosterone (**E**) in TM3 cells. The effects of PCA on survival rate (**F**) and expression of testosterone (**G**) and antioxidant enzymes (**H**) in TM3 cells induced by AAPH. *CTL* Control group, *PCAL* Basic diet containing 500 mg/kg PCA group, *PCAH* Basic diet containing 1,000 mg/kg PCA group. *HO-1* Heme oxygenase-1, *NQO1* NADH quinone oxidoreductase 1, *SOD2* Superoxide dismutase 2. Data were shown as mean ± SD (*n* = 6). ^*^P < 0.05, ^**^P < 0.01, ^***^P < 0.001, ^****^P < 0.0001

Testosterone is a crucial hormone for maintaining spermatogenesis. As shown in Fig. 3, the addition of 1,000 mg/kg of PCA significantly increased serum testosterone levels in boars (*P* < 0.05) (Fig. 3A). Adding PCA significantly enhanced total antioxidant capacity (T-AOC) in boar serum (*P* < 0.05) (Fig. 3B). The addition of 1,000 mg/kg of PCA significantly reduced serum malondialdehyde (MDA) levels (*P* < 0.05) (Fig. 3C). Leydig cells in the testicular interstitium are the primary cells responsible for testosterone synthesis. To investigate whether PCA improves antioxidant capacity and restores testosterone levels in boars, an oxidative damage model was established using TM3 Leydig cells line. The addition of 15, 20, and 25 mmol/L of AAPH treatment dramatically decreased the cell survival rate and 10, 15, 20, and 25 mmol/L of AAPH treatment significantly reduced testosterone production in TM3 cells (Fig. 3D and E). Thus 15 mmol/L AAPH treatment was used to make an oxidative stress model. Following AAPH induction, the addition of 30, 60, and 90 μmol/L of PCA restored testosterone production in TM3 cells and alleviated oxidative stress-induced cell apoptosis (*P* < 0.05) (Fig. 3F and G). Western blot analysis revealed that under oxidative stress conditions, 30 μmol/L of PCA restored the expression of antioxidant proteins HO-1, SOD2, and NQO1 to control levels. In comparison, 60 and 90 μmol/L promoted the expression of HO-1, SOD2, and NQO1 antioxidant proteins.

### Protocatechuic acid reduces sperm reactive oxygen species (ROS) and improves mitochondrial function

**Fig. 4:**
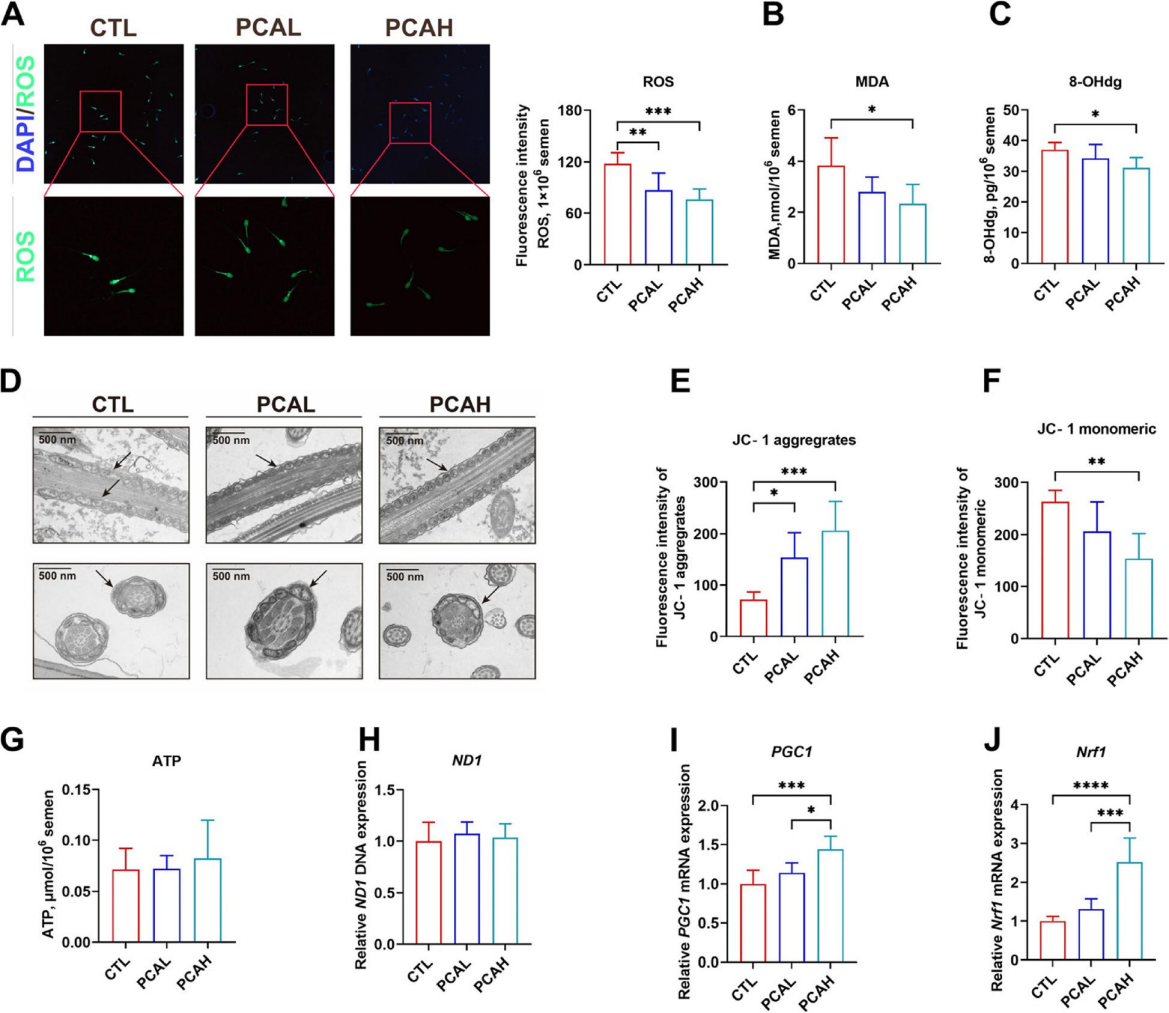
The impact of protocatechuic acid (PCA) on the distribution of reactive oxygen species (ROS), the content of ROS (**A**), the content of malondialdehyde (MDA) (**B**), and the content of 8-hydroxy-2'-deoxyguanosine (8-OHdg) (**C**) in sperm is examined. The effects of PCA on the morphology (**D**), membrane potential (**E** and **F**), ATP content (**G**), and the DNA expression of *ND1* in sperm (**H**). The influence of PCA on the mRNA expression of peroxisome proliferator-activated receptor *PGC1* (**I**) and *Nrf1* (**J**) in sperm. *CTL* Control group, *PCAL* Basic diet containing 500 mg/kg PCA group, *PCAH* Basic diet containing 1,000 mg/kg PCA group. Data were shown as mean ± SD (*n* = 6). ^*^P < 0.05, ^**^P < 0.01, ^***^P < 0.001, ^****^P < 0.0001

Sperm are highly susceptible to ROS attack, leading to sperm defects and functional impairments. As shown in Fig. 4A, both the addition of 500 and 1,000 mg/kg of PCA can reduce sperm ROS levels compared to the control group (*P* < 0.01). Furthermore, the addition of 1,000 mg/kg PCA significantly decreased the levels of lipid oxidation marker MDA (Fig. 4B) and DNA oxidative damage marker 8-OHdg (Fig. 4C) in sperm (*P* < 0.05). Sperm vitality relies on mitochondrial function, and mitochondria located on the sperm flagella may provide energy for sperm motility. Fluorescent quantitative PCR analysis of *ND1* revealed that adding PCA did not increase the quantity of sperm mitochondria (Fig. 4H). The addition of PCA had no significant effect on sperm ATP content (Fig. 4G). However, transmission electron microscopy scanning of sperm flagellar mitochondria showed that the control group exhibits morphological heterogeneity with swollen mitochondrial cristae. In contrast, the PCAH group demonstrated normal mitochondrial morphology with some swollen cristae. The PCAH group exhibited well-arranged flagellar mitochondria. Although some mitochondrial cristae were swollen, the overall structure of mitochondria remained intact (Fig. 4D). JC-1 staining was used to assess mitochondrial membrane potential. After adding 500 and 1,000 mg/kg PCA, there was a significant increase in JC-1 aggregates (*P* < 0.01). The addition of 1,000 mg/kg significantly decreased in JC-1 monomers (*P* < 0.01) (Fig. 4E and F), indicating that the addition of PCA can enhance the mitochondrial membrane potential (ΔΨm) of sperm. Additionally, the addition of PCA increased the transcriptional expression of mitochondrial biogenesis regulatory factors *PGC1* (*P* < 0.05) and *Nrf1* (*P* < 0.01) (Fig. 4I and J).

### PCA can selectively bind to and activate AMPK

**Fig. 5:**
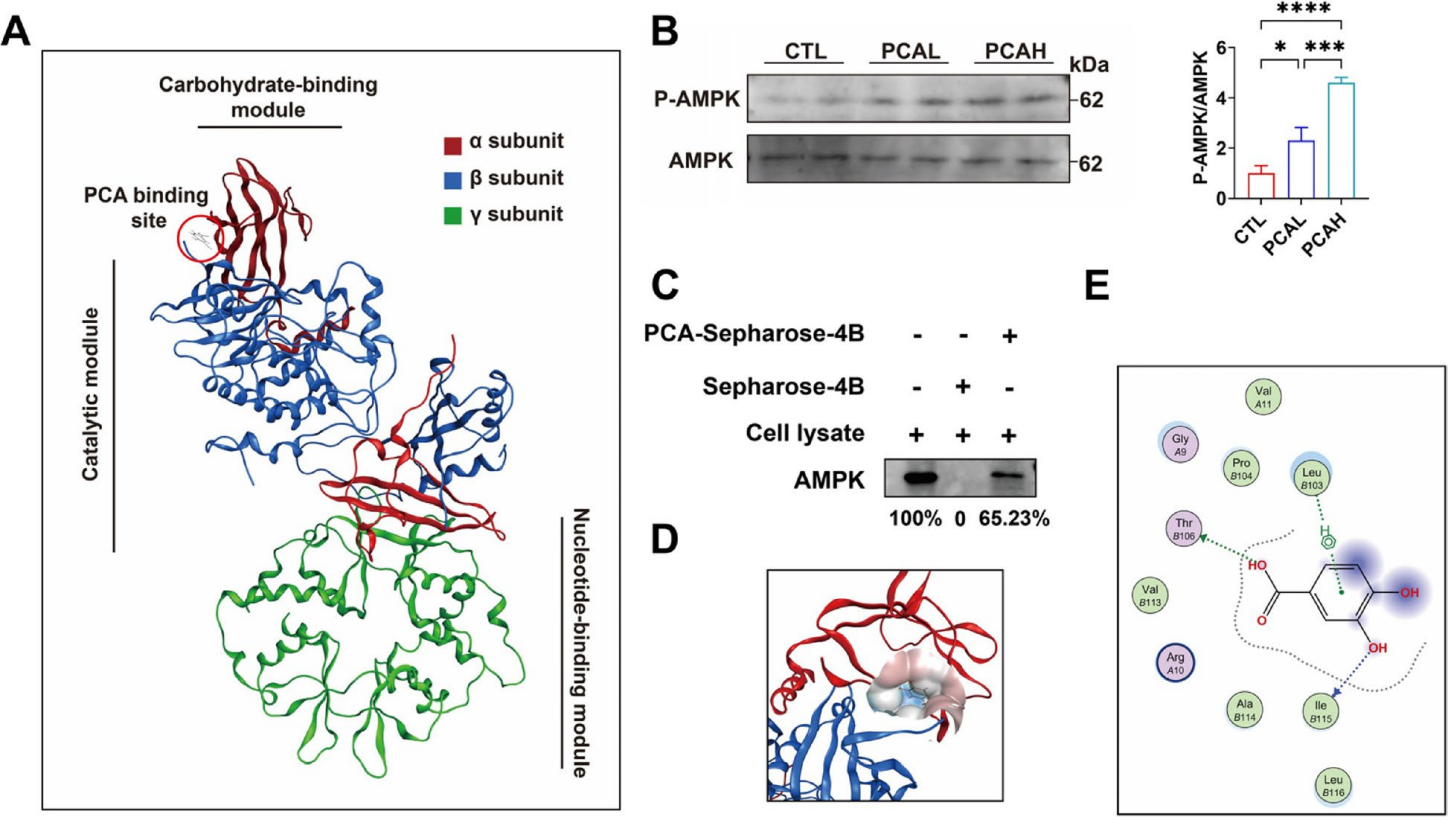
Binding ability and regulation of resveratrol to adenosine 5′-monophosphate (AMP)-activated protein kinase (AMPK) and docking model between protocatechuic acid (PCA) and AMPK. The docking models of resveratrol to AMPK (**A**, **D**, **E**). The protein expression level of AMPK and phosphorylated AMPK (p-AMPK) and the binding abilities of PCA to AMPK was detected by immunoblotting (**B** and **C**)

The regulation of mitochondrial function is primarily attributed to AMPK. We found that both the PCAL and PCAH groups activated sperm AMPK, promoting its phosphorylation (Fig. 5B). Pull-down results revealed that PCA can bind to AMPK, with a binding rate of 65.23% (Fig. 5C). AMPK exists as a trimeric complex composed of a catalytic subunit (α subunit) and two regulatory subunits (β and γ subunits) (Fig. 5A). The N-terminal region of the α subunit comprises the kinase domain (KD). Full activation of AMPK requires phosphorylation of the conserved threonine residue in the KD activation loop (commonly referred to as Thr172). Molecular modeling studies revealed that PCA can bind to Thr B106 and Ile B115 within the KD structure of the α subunit (Fig. 5E), forming a pocket structure in the C-lobe (Fig. 5D). This binding site has been identified as the binding site for many direct AMPK activating compounds.

## Discussion

The duration of boar utilization significantly influences a swine farm’s productivity and economic benefits. During the 1–2 years old, the primary phase of culling in boars significantly restricts the manifestation of their reproductive potential. The annual culling rate of boars can reach a substantial 59.4%, exacerbating the utilization of young boars and elevating the probability of reproductive disorders, ultimately culminating in a reduction in the duration of boar utilization [24]. The principal factor leading to boar culling is poor semen quality, which diminishes the boar’s lifespan by approximately 12.3 months [24]. In a natural setting, boars achieve their maximum sperm production capacity at 42 months of age. Theoretically, boars can continue being used for breeding until they reach 48 months of age. However, boars are often culled at an age significantly younger than this theoretical threshold in practical production settings. Several initial studies have systematically examined the alterations in boar semen quality as they age. Clark et al. [3] reported that a significant increase in the average total sperm count of boars from 8 to 10 months up to 14 months, followed by minimal change. Similarly, Smital [4] noted a rapid increase in sperm production with advancing age of boars, although their study found that maximum production occurred at a relatively later age (3.5 years). Furthermore, semen volume continuously increased until approximately 2 years of age, after which it remained relatively stable. In a study conducted by Wolf et al. [25], it was observed that boars exhibited a substantial increase in sperm concentration prior to reaching 11 months of age, which was subsequently followed by a moderate decline until the age of 3 years, at which point it reached a stable state. Concurrently, there was a consistent decrease in sperm motility as age progressed. Conversely, the total sperm count and the quantity of functional sperm experienced an initial dramatic increase, culminating in their highest levels at approximately 2 years of age [25]. This study selected a sample of 38 PIC boars, and the findings were consistent with previous research. Starting from 9 months of age, when the boars were introduced to breeding, there was a progressive increase in sperm density, total sperm count, sperm motility, and ejaculate volume, with peak values observed at 18–20 months of age. However, a noteworthy deviation was observed at 20 months of age, where a decline in these parameters became evident. By 37 months of age, sperm density, total sperm count, sperm motility, and ejaculate volume were significantly lower compared to the earlier peak values.

According to the findings above, a notable decrease in key sperm parameters was observed in boars beyond the age of 28 months. The decline in semen quality can be attributed to a range of factors, such as obesity, oxidative stress, systemic inflammation, and excessive frequency of ejaculation. In the present study, PCA, a polyphenol renowned for its lipid-lowering, anti-inflammatory, and potent antioxidant attributes, was chosen as the subject of investigation to assess its impact on the quality of boar semen. Considering that the boar's spermatogenesis cycle spans 42 d, our experimental design encompassed 84 d, equivalent to two complete spermatogenesis cycles [26]. The findings of this study indicate that the inclusion of PCA in the feed resulted in a significant enhancement of sperm motility and viability while also mitigating the observed decrease in concentration of spermatozoa, total number of spermatozoa, and semen volume. These results support the notion that incorporating PCA into the diet positively influences the quality of boar semen.

Testosterone plays a crucial role in the sustenance of spermatogenesis, with the testes necessitating a substantial amount of testosterone to facilitate the customary course of sperm production. The present study observed a notable elevation in serum testosterone levels in boars upon the intervention of PCA. In steroidogenic cells, the generation of ROS is notably heightened, as both the mitochondrial electron transport chain and cytochrome P450 enzymes implicated in steroid hydroxylation can generate ROS [27]. Research findings indicate that elevated levels of ROS in the testes of elderly mice contribute to a decline in steroidogenic enzymes, ultimately leading to diminished steroid production [28]. In Leydig cells, aging is associated with decreased in antioxidant defense molecules, including superoxide dismutase-1 and -2, glutathione peroxidase, and reduced glutathione (GSH) [29]. In the present study, it was observed that the administration of PCA resulted in a significant increase in the overall antioxidant capacity of serum and a reduction in the levels of MDA, as well as the restoration of testosterone secretion. This observed effect is likely attributed to the antioxidant properties exhibited by PCA. Furthermore, through the assessment of sperm ROS levels and indicators of oxidative damage, it was discovered that PCA can diminish ROS content in sperm and alleviate lipid peroxidation and protein oxidation, as levels of markers associated with DNA oxidative damage. During the maturation, the sperm experiences a comprehensive reorganization of its membrane structures within the epididymis [30]. As the sperm progresses from the head to the tail of the epididymis, there is a notable augmentation in both membrane fluidity and the presence of polyunsaturated fatty acids (PUFAs) [31, 32]. Nevertheless, it is essential to note that PUFAs within cell membranes are particularly vulnerable to oxidative harm [31]. Consequently, it can be inferred that by bolstering the body's antioxidant capabilities, PCA may mitigate the deterioration in sperm quality. In order to further substantiate the impact of PCA, we opted for the TM3 Leydig cell line and employed an AAPH-induced oxidative stress model. The stimulation of AAPH resulted in a notable inhibition of testosterone synthesis in TM3 cells. Nevertheless, the introduction of PCA not only reinstated the apoptosis of TM3 cells under AAPH stimulation, but also reinstated the secretion of testosterone by Leydig cells. Several dietary polyphenols have been reported to trigger the expression of phase II anti-oxidant enzymes [33]. Under the induction of AAPH, the administration of PCA resulted in increased levels of SOD2, HO-1, and NQO1 in TM3 cells. This suggests that PCA can potentially mitigate the reduction in testosterone synthesis and oxidative damage to sperm by augmenting the levels of phase II stress response enzymes SOD2, HO-1, and NQO1. Unfortunately, this study did not include a control group of healthy boars with normal sperm quality, which hinders the investigation of whether the decline in semen quality is attributable to oxidative stress within the organism.

The findings of this study demonstrate that PCA can potentially augment sperm motility, which pertains to the sustained movement of sperm through the propulsion of its flagellum. Mitochondria assume a pivotal role as the principal supplier of energy for sperm vitality, as the oxidative phosphorylation mechanism within mitochondria is relied upon by sperm to produce the requisite ATP for its locomotion [34]. Consequently, the effective operation of mitochondria is imperative for attaining optimal sperm vitality. The present study did not detect any elevation in ATP levels in sperm after the intervention of PCA. Furthermore, there was no augmentation in the quantity of sperm mitochondria. Throughout spermiogenesis, in sperm cell differentiation, a portion of mitochondria and the majority of cytoplasm are eliminated in the residual body [35]. The remaining mitochondria undergo reorganization and accumulate in a spiral manner within a slender tubular structure around the flagellum's anterior region [35]. In the present study, noteworthy deviations in the ultrastructural composition of sperm flagellar mitochondria were observed in the control group, encompassing cristae swelling, disarrayed organization, and vacuolization. Nevertheless, the intervention of PCA exhibited a partial amelioration of the aforementioned structural impairments in mitochondria, thereby suggesting its capacity to alleviate the deleterious alterations in both mitochondrial structure and functionality linked to diminished sperm vitality. The flagellum is tightly enveloped by the mitochondrial sheath, crucial in optimizing ATP production and energy transport efficiency, directly influencing sperm motility [36]. The observed enhancements in mitochondrial structure following PCA treatment indicate a potential mechanism for augmenting sperm motility through optimizing ATP generation and energy transfer within the mitochondrial sheath. The results of this study demonstrate that PCA can alleviate mitochondrial vacuolization and abnormalities while also enhancing the mitochondrial membrane potential, as evidenced by the JC-1 staining of spermatozoa. These findings suggest that PCA positively affects on sperm motility by mitigating mitochondrial dysfunction. Additionally, our investigations have unveiled that PCA induced upregulation in the expression of pivotal genes implicated in facilitating mitochondrial biogenesis, specifically *PGC1* and *Nrf1*. Traditionally, *PGC1* has been recognized as an indispensable gene in promoting mitochondrial biogenesis; nevertheless, recent studies have demonstrated that the elimination of *PGC1* results in significant disturbances in both the structure and function of mitochondria [37]. *Nrf1* serves as an initial transcription factor regulated and induced by *PGC-1* to facilitate the process of mitochondrial biogenesis [38]. Furthermore, *Nrf1* can form an activating complex with PGC1α, thereby enhancing the transcription of ferredoxin reductase and contributing to the maintenance of mitochondrial function [38, 39]. Both the transcription of *PGC-1* and *Nrf1* are subject to the control of AMPK, which was found to be activated by PCA in the present study [40]. The activation of AMPK by PCA may play a pivotal role in coordinating the regulation of *PGC-1* and *Nrf1*, consequently promoting mitochondrial biogenesis and augmenting mitochondrial function. AMPK is a heterotrimeric complex consisting of a catalytic α subunit, regulatory β, and γ subunits. In vitro sedimentation assays have demonstrated the strong affinity of PCA for AMPK [41]. Molecular simulations have further elucidated that PCA can effectively bind to the α subunit within the pocket formed by the KD and the C-terminal lobe, explicitly interacting with Thr B106 and Ile B115. This binding site has been identified as the common site for numerous direct AMPK activators [42]. This study's results indicate a potential direct interaction between PCA and AMPK, leading to the modulation of AMPK activity. This interaction may play a role in the observed impact of PCA on mitochondrial biogenesis and function in sperm cells.

## Conclusion

The semen volume, concentration and total number of spermatozoa in boars exhibited an increase from 9 to 19 months, and show significant linear decreased trend in 28, 24, and 22 months old. Sperm motility displayed an upward trajectory, reaching its peak at 20 months of age, and show significant linear decreased trend in 20-month-old. Dietary supplementation of PCA demonstrated the ability to mitigate the decrease in semen volume, concentration of spermatozoa, total number of spermatozoa, and significantly increase the sperm motility and increase the sperm viability. Moreover, PCA possesses the ability to mitigate oxidative damage in testicular stromal cells by upregulating the expression of HO-1, SOD2, and NQO1. PCA can restore serum testosterone levels and alleviate oxidative damage in sperm. Additionally, PCA exhibits the capacity to enhance phosphorylation by selectively binding to AMPK protein, thereby improving sperm mitochondrial function and augmenting sperm motility via *PGC-1*/*Nrf1*.

### Supplementary Information

**Additional file 1:** **Table S1. **GenBank accession numbers, sequences of forward and reverse primers, and fragment sizes used for Real-Time PCR. **Table S2. **Antibody information and incubation conditions.

## Data Availability

All data generated or analyzed during this study can be made available by the corresponding author upon reasonable request.

## Abbreviations

AAPH: 2,2′-Azobis-2-methyl-propanimidamide
AMPK: Adenosine 5′-monophosphate-activated protein kinase
C3G: Cyanidin-3-O-glucoside
GSH: Glutathione
HO-1: Heme oxygenase-1
KD: Kinase domain
MDA: Malondialdehyde
NQO1: NADH quinone oxidoreductase 1
Nrf1: Nuclear respiratory factor 1
NADPH: Nicotinamide adenine dinucleotide phosphate
ND-1: NADH dehydrogenase-1
PIC: Pig Improvement Company
PGC-1: Peroxisome proliferator-activated receptor-γ coactivator-1
PCA: Protocatechuic acid
qPCR: Quantitative polymerase chain reaction
ROS: Reactive oxygen species
SOD2: Superoxide dismutase 2
T-AOC: Total antioxidant capacity
