# Supplementary material for: Patterns of alteration in boar semen quality from 9 to 37 months old and improvement by protocatechuic acid
Source: J Anim Sci Biotechnol. 2024 May 17;15:78. doi: 10.1186/s40104-024-01031-6 (PMC11100174; doi:10.1186/s40104-024-01031-6)
Supplement: Supplementary file 1 — Additional file 1: Table S1. GenBank accession numbers, sequences of forward and reverse primers, and fragment sizes used for Real-Time PCR. Table S2. Antibody information and incubation conditions. [file 40104_2024_1031_MOESM1_ESM.docx]

**Additional file 1**

**Table S1** GenBank accession numbers, sequences of forward and reverse primers, and fragment sizes used for Real-Time PCR

| **Target** | **GeneBank number** | **Primer sequence** | **Size, bp** |
| --- | --- | --- | --- |
| GADPH | NM 001206359.1 | F: 5' GGCTGTGGGCAAGGTCATCC 3'  R: 5' GTTTCTCCAGGCGGCAGGTC 3' | 110 |
| β-actin | XM 021086047.1 | F: 5' TCTGGCACCACACCTTCTACAAC 3'  R: 5' CACTGGAAGGCGAAGGTTT 3' | 106 |
| Nrf1 | XM 021079000.1 | F: 5' GTTTCATGGACCCAGGCACTACG 3'  R: 5' TGGTTGTGGCTTGTGTTTGTGTTTG 3' | 115 |
| PGC1α | XM 054348813.1 | F: 5' CGATGACCCTCCTCACACCAAAC 3'  R: 5' TTGCGACTGCGGTTGTGTATGG 3' | 97 |
| ND1 | NC 000845.1 | F: 5' AGCCATGTCAAGCCTAGCAGTC 3'  R: 5' GCCCCGATGAGTGCGTATTTTG 3' | 78 |
| GCG | NC 010457.5 | F: 5' GAATCAACACCATCGGTCAAAT 3'  R: 5' CTCCACCCATAGAATGCCCAGT 3' | 97 |

| **Antibodies** | **Cat. No.** | **Diluted ratio** | **Loading quantity of protein sample** | **Incubation conditions** | **Exposure time** |
| --- | --- | --- | --- | --- | --- |
| Phospho-AMPK | Thermo Fisher PA5-17831 | 1: 200 | 40 μg | 4 °C overnight | 120 s -180 s |
| AMPK | Abcam ab231807 Abcam | 1: 500 | 40 μg | 4 °C overnight | 60 s-100 s |
| HO-1 | CST# 43966S | 1: 1,000 | 10 μg | 4 °C overnight | 10 s-30 s |
| SOD2 | CST# 13141S | 1: 1,000 | 10 μg | 4 °C overnight | 10 s-30 s |
| NQO1 | CST# 62262S | 1: 1,000 | 10 μg | 4 °C overnight | 10 s-30 s |
| β-Actin | CST# 8457S | 1: 2,000 | 10 μg | 4 °C overnight | 4 s -8 s |
| HRP-linked Antibody | CST# 7074 | 1: 5,000 | - | Room temperature for 1 h | - |

**Table S2** Antibody information and incubation conditions
